# Nutritional status of preschoolers in the public school system of Macaé: a panel study conducted between 2012-2014 and 2017-2019 in the context of the oil crisis and the implementation of educational actions based on the Dietary Guidelines for the Brazilian Population

**DOI:** 10.1590/S2237-96222025v34e20240386.en

**Published:** 2025-08-04

**Authors:** Ana Eliza Port Lourenço, Thácia de Araújo Amado Coutinho, Luana Silva Monteiro, Laís Vargas Botelho, Priscila Vieira Pontes, Naiara Sperandio

**Affiliations:** 1 Universidade Federal do Rio de Janeiro, Instituto de Alimentação e Nutrição, Macaé, RJ, Brazil

**Keywords:** Anthropometry, Preschool children, Epidemiological Surveys, Dietary Guidelines, School Health Services, Antropometría, Preescolar, Encuestas Epidemiológicas, Guías Alimentarias, Servicios de Salud Escolar

## Abstract

**Objective:**

To assess the nutritional status of preschool children in the public school system of Macaé, Rio de Janeiro, at two apart times, separated by an interval of five years.

**Methods:**

This is a panel study conducted between 2012-2014 and 2017-2019, including five schools, located in regions with different socioeconomic conditions. The samples covered 15.0% of the total number of students in the public early childhood education network in Macaé. Indicators of height-for-age and body mass index for age were evaluated. Summary measurements of anthropometric variables were calculated, with stratification by sex and age group. For comparisons between groups and surveys, Student’s t-test or Mann-Whitney and chi-square tests were used.

**Results:**

1,028 and 1,005 preschoolers were evaluated in each period. The average age was 55.3 and 54.7 months, with 50.6% and 51.6% being girls. Height deficit was identified in 1.9% and 2.1% of the children, with no significant difference by sex, age group or between surveys. The prevalence of obesity was 5.1% and 4.7% among those under 5 years old, and 10.1% and 11.0% among the oldest, also with no difference between the surveys.

**Conclusion:**

At both moments of the panel, the prevalence of nutritional deficit was low, and of obesity, relatively high. Despite the context of crisis in the local economy, the frequency of anthropometric indicators did not deteriorate between the surveys. The implementation of educational actions in schools, guided by the Dietary Guidelines for the Brazilian Population, may have contributed to protecting children’s health.

## Resumen

**Objetivo:**

Evaluar el estado nutricional de niños preescolares de la red pública de enseñanza de Macaé, Rio de Janeiro, en dos momentos diferentes, separados por un intervalo de cinco años.

**Métodos:**

Se trata de un estudio de panel realizado entre 2012-2014 y 2017-2019, incluyendo cinco escuelas, ubicadas en regiones con diferentes condiciones socioeconómicas. Las muestras abarcaron el 15% del total de alumnos de la red pública de educación infantil de Macaé. Se evaluaron los indicadores talla-para-la-edad e índice de masa corporal-para-la-edad. Se calcularon medidas de resumen de las variables antropométricas, con estratificación por sexo y grupo etario. Para las comparaciones entre grupos y encuestas se utilizó la prueba t de Student o la prueba de Mann-Whitney y la prueba de chi-cuadrado.

**Resultados:**

En cada período se evaluaron 1.028 y 1.005 preescolares. La edad media fue de 55,3 y 54,7 meses, siendo el 50,6% y 51,6% niñas. Se identificó déficit de talla en el 1,9% y el 2,1% de los niños, sin diferencias significativas por sexo, grupo de edad o entre encuestas. La prevalencia de obesidad fue de 5,1% y 4,7% entre los menores de 5 años, y de 10,1% y 11% entre los mayores, también sin diferencias entre las encuestas.

**Conclusión:**

En ambos momentos del panel, la prevalencia de déficit nutricional fue baja y la de obesidad, relativamente alta. A pesar del contexto de crisis en la economía local, la frecuencia de los indicadores antropométricos no se deterioró entre momentos. La implementación de acciones educativas en las escuelas, guiadas por la *Guía Alimentaria para la Población Brasileña*, puede haber contribuido a la protección de la salud infantil.

**Ethical aspects**

This research respected ethical principles, having obtained the following approval data:

Research Ethics Committee: Universidade Federal do Rio de Janeiro

Opinion number: 1.319.805

Approval date: 17/11/2015

Certificate of Submission for Ethical Appraisal: 49258313.1.0000.5257

Informed Consent Form: Obtained from all participants prior to collection of surveys, from 2016 to 2019.

Observations: The data from the survey conducted from 2012 to 2014 were curated by the Municipal Department of Education of Macaé and were provided to Professor Ana Eliza Port Lourenço for study.

## Introduction

Nutritional status can be understood as a summary of the relationship between individuals, food, and the environment. When there is an imbalance in this relationship, nutritional deviations occur that pose health risks (1). Such an imbalance may occur due to the mosaic of determinants, both proximal and more distal to individuals, which interfere in the nutritional transition process in societies (2). Monitoring the nutritional status of communities is essential, especially in childhood, as it allows us to assess children’s growth, weight gain and development, in addition to helping to target health-related policies, programs and actions (3).

Anthropometric indicators are useful, as they identify nutritional deviations and reflect the general health conditions of societies (4). Food and nutritional surveillance in Primary Healthcare and research to monitor the nutritional transition in Brazil exemplify the potential application of these indicators (5-7). It is also pertinent to use them in studies, on a large or smaller scale, that aim to evaluate trends based on political and social developments, such as the emergence of food classification according to its nature, extent and purpose of processing, expressed in the Dietary Guidelines for the Brazilian Population (8); or unexpected circumstances, such as the COVID-19 pandemic (9) and other crisis situations.

Small-scale research is relevant, as events at a global or national level can have different repercussions on different local situations. The smaller scale allows for the analysis of details of the nutritional context that might not be noticeable from a macro perspective. The global crisis in the oil industry, which occurred in 2014, had a drastic impact on Brazilian municipalities in this economic base, such as Macaé, located in the north of the state of Rio de Janeiro. Macaé has one of the highest per capita gross domestic product values in the country; however, because of the crisis, there was disinvestment in the municipality, with losses to health and quality of life (10,11).

Another particularity of Macaé is that, despite being a medium-sized municipality, relatively far from the capital city, includes four public universities. The liveliness of university research and extension projects favors the construction of links with local services and society. Specifically, projects on the theme of health promotion and healthy eating stand out (12-14).

Since its publication in 2014, the Dietary Guidelines for the Brazilian Population has become the reference for these projects, which operate in different spaces, especially schools, as they are favorable places for the materialization of intersectoral actions, bringing health and education closer together (15). The recommendations of these Dietary Guidelines began to be applied in line with the Brazilian National School Feeding Program (PNAE) and the Health at School Program, which favored healthy habits among children and educators (16). 

The objective of this study was to evaluate the nutritional status of preschool children from the public school system in Macaé, Rio de Janeiro, at two apart times, separated by an interval of five years. It was hypothesized that, due to the oil crisis, there would be a worsening in the frequency of different forms of child malnutrition.

## Methods

### Study design

This is an observational panel study that took place in two moments: from May 2012 to July 2014 (moment 1) and from May 2017 to December 2019 (moment 2). The study analyzed primary data collected in public early childhood education schools, through a partnership between the Federal University of Rio de Janeiro and the municipal education service of Macaé. The anthropometric assessment interval for moments 1 and 2 was approximately five years in each school.

### Context: university-service articulation in the operationalization of the PNAE

The municipal public education network of Macaé had 107 schools, which served 40,000 students (17). The School Nutrition Coordination of the Municipal Department of Education of Macaé was responsible for implementing the PNAE at the municipal level. In addition to providing food to students, the PNAE provides for anthropometric assessment and the implementation of food and nutritional education activities, based on nutritional diagnosis (18). 

During the period of this study, the School Nutrition Coordination team was made up of four nutritionists, an insufficient number to meet the demands of the network and fully operationalize the PNAE guidelines. The management of school meals and menu planning were conducted by the coordination, in accordance with current legislation, while the execution of the menus was the responsibility of a third-party company. Anthropometric and educational activities were scarce. To address this limitation and enhance the coverage of actions, a partnership was established between the coordination and a study center of the aforementioned university (14), which has worked in an integrated manner with the service since 2011.

This articulation occurred through extension projects of this center, which collaborated directly in the service routine, aiming to achieve the goals set by the PNAE. This study is a result of this university-service approach, in which teachers and undergraduates join forces with nutritionists from the municipality, forming a single team. Although it is a primary study, data collection followed the modus operandi usual of the School Nutrition Coordination. The field logistics and variables collected were not specifically defined to meet the objectives of a study. On the contrary, conducting this study represented the achievement of the objectives of the coordination and, ultimately, of the PNAE. 

The following actions were carried out in each school: a planning visit; two to three visits for anthropometric measurements; a visit to share results and plan educational activities; and visits to conduct activities, two to three with the children and one to two with educators and families, depending on the size of the school. In addition to the coordination team and the university, healthcare professionals from the assigned territory collaborated in educational activities, integrating with the Health in School Program. 

The actions were guided by the Food and Nutrition Education Reference Framework for Public Policies (19) and the Dietary Guidelines (8). The educational methodology used is detailed in previous publications (13,14,20). In general, the activities with children used the guidelines of the Dietary Guidelines in a playful way to address avoiding ultra-processed foods; basing the diet on in natura or minimally processed foods; and developing culinary skills. With educators and families, in addition to general recommendations of the Dietary Guidelines, the actions aimed to promote reflection on the principle of autonomy in food choices.

### Data sources and measurement

Data from moments 1 and 2 were collected within the scope of the School Nutrition Coordination service, in partnership with the university’s study center. The center provided the equipment for data collection and prior training for the team, which included nutritionists from the coordination and university students. 

At both times, the anthropometric assessment took place as provided for in Brazilian Primary Healthcare (7), during school hours, with the children dressed in their school uniform. The weight was measured in kilograms, using an electronic scale, Tanita brand, with an accuracy of 100 g. Height was measured in centimeters, twice consecutively for each child, using a wooden stadiometer, Alturaexata brand, with 1 mm precision. For analysis purposes, the average of the two measurements was used. Information about gender and date of birth was obtained from school documents. Age was calculated by the difference between the measurement and birth dates.

### Participants

At both surveys, a non-probabilistic sample was evaluated, consisting of preschool children aged 2 to 7 years old, enrolled in five schools in urban areas. These were selected for convenience by the School Nutrition Coordination, which, due to logistical difficulties, prioritized units with a larger number of students and easy access by public transport. 

In the urban area of Macaé, there were 45 public early childhood education schools, totaling 8,000 preschoolers. The city is subdivided into administrative sectors, which can be grouped into three socioeconomic regions. Two of the schools evaluated were in the poorest region, where there is disorderly urban growth and poor sanitation. Another school was in the intermediate socioeconomic region, characteristic of the center of Macaé. The other two were on the coastal area, characterized by a variety of commercial establishments, tourism, and high-standard residences.

As inclusion criteria, all preschoolers regularly enrolled in the selected schools were considered. Students with mental or physical health conditions that interfered with the anthropometric assessment were excluded. 

At moment 1, of the 1,209 preschoolers enrolled, 24 were excluded and 157 were lost, with 1,028 preschoolers being analyzed (85.0% of those eligible). At moment 2, of the 1,116 children enrolled in the same schools, 8 were excluded, 103 were lost and 1,005 were analyzed (90.0% of those eligible).

### Variables 

The numerical variables analyzed were those provided for in the PNAE (weight, height, and age), as well as the anthropometric indices of height-for-age and body mass index-for-age. These indices were expressed in z-scores, calculated according to the World Health Organization (WHO) reference curves (4.21), using the WHO Anthro Plus program (v1.0.4).

Such indexes were also categorized. Children with z-score <-2 standard deviations for height-for-age or body mass index-for-age were considered at deficit in these indicators. Children under 5 years of age were classified as overweight when the body mass index-for-age was ≥2 standard deviations and <3 standard deviations, and with obesity, when it was ≥3 standard deviations. Older preschoolers were considered overweight when body mass index-for-age was ≥1 standard deviation and <2 standard deviations, and with obesity, when it was ≥2 standard deviations (7). 

### Bias control

The data collection procedures were the same at both times, as were the indicators analyzed, which favored comparison. To avoid losing participants, more than one visit was made to each school, on different and non-consecutive days of the week.

To minimize errors, information on sex and date of birth recorded in school documents was used. Height measurement was performed in duplicate. 

Because it is not probabilistic, the sample could suffer from selection bias. By including schools from different socioeconomic regions, we sought to mitigate this bias and improve external validity.

### Study size 

At both times, the number of children in the sample covered approximately 15.0% of the total number of preschoolers in the urban area and those studying in each of the three socioeconomic regions of Macaé.

### Statistical methods

A descriptive analysis of anthropometric variables and nutritional deviations was performed according to period (moment 1, moment 2), sex (male, female) and age group, considering preschoolers under 5 years of age and those aged 5 years or older. Categorical variables were summarized in absolute and relative frequencies, and continuous variables in mean and standard deviations or medians and interquartile range, depending on the data distribution. Between-group comparisons for continuous variables were performed using Student’s t-test or Mann-Whitney test. For categorical variables, the chi-square test was used. 

Power analyses were conducted to assess the study’s ability to identify real differences in the prevalence of nutritional deviations between the two panel moments, in each age group, considering a minimum statistical power of 80.0%. It was observed that the sample obtained at both times had a statistical power of 100.0% to detect the prevalence of overweight and obesity among children under 5 years of age and among older children. However, the sample power was not capable of detecting differences in the frequencies of height-for-age and body mass index-for-age deficits.

A significance level of 5.0% was adopted in all analyses. The Statistical Package for the Social Sciences, version 19, was the statistical program used.

## Results

Among the 1,028 children assessed at moment 1, 50.6% were female, 59.5% were under 5 years old, and the overall average age was 55.3 months. Among the 1,005 participants at time 2, 51.6% were girls, 61.2% were under 5 years old, and the overall mean age was 54.7 months (Tables 1 and 2).

The height-for-age means were close to 0 (Table 2). The distribution curve resembled the WHO reference at moment 1 and 2. Among boys, the height-for-age distribution showed lower values at moment 2 (p-value 0.020). The prevalence of height-for-age deficit was 1.9% at moment 1 and 2.1% at moment 2, with no significant difference according to sex, age group or between time points (Table 1). 

**Table 2:** Descriptive statistics of anthropometric variables and age, according to age group and sex of preschool children in the public school system. Macaé, 2012-2014 (n=1,028) and 2017-2019 (n=1,005)

	2012-2014					2017-2019				
	Age (months)	Height (cm)	Weight (kg)	Height-for-age (z-score)	Body mass index-for-age (z-score)	Age (months)	Height (cm)	Weight (kg)	Height-for-age (z-score)	Body mass index-for-age (z-score)
	Mean (standard deviation)	Median (interquartile range)				Mean (standard deviation)	Median (interquartile range)			
**Children <5 years**	46.5 (8.8)	102.5 (97.5; 106.8)	16.7 (14.9; 19.0)	-0.02 (-0.71; 0.77)	0.61 (-0.15; 1.39)	46.0 (8,9)	101.1 (95.8; 106.8)	16.4 (14.6; 18.6)	-0.12 (-0.86; 0.63)	0.56 (-0.04; 1.26)
**Children ≥5 years**	68.2 (4.9)	114.1 (110.3; 118.0)	20.4 (18.7; 23.0)	0.13 (-0.54; 0.78)	0.28 (-0.39; 1.06)	68.4 (5.0)	113.8 (110.3; 117.5)	20.5 (18.6; 22.9)	-0.01 (-0.61; 0.71)	0.44 (-0.31; 1.11)
Girls	55.3 (12.8)	106.8 (100.4; 113.4)	18.2 (15.6; 20.9)	-0.02 (-0.60; 0.71)	0.47 (-0.31; 1.21)	54.4 (13.3)	106.9 (98.9; 112.0)	18.0 (15.4; 20.3)	-0.10 (-0.74; 0.64)	0.45 (-0.21; 1.14)
Boys	55.3 (13.2)	107.9 (101.5; 113.9)	18.6 (16.4; 21.1)	0.12^a^ (-0.64; 0.83)	0.52 (-0.27; 1.34)	55.0 (13.3)	107.3 (100.0; 114.0)	18.3 (16.0; 21.2)	-0.04^a^ (-0.82; 0.68)	0.57 (-0.03; 1.24)
Total	55.3 (13.0)	107.4^a^ (100.9; 113.6)	18.3 (16.0; 21.0)	0.03^a^ (-0.62; 0.77)	0.48 (-0.28; 1.29)	54.7 (13.3)	107.1^a^ (99.4; 112.9)	18.1 (15.7; 20.8)	-0.08^a^ (-0.78; 0.66)	0.52 (-0.14; 1.20)

^a^The distribution of the variable differs significantly between periods.

**Table 1:** Prevalence of nutritional deviations, according to age group and sex of preschool children in the public school system. Macaé, 2012-2014 (n=1,028) and 2017-2019 (n=1,005)

	2012-2014					2017-2019				
	Short height	Low weight	Overweight	Obesity	Total	Short height	Low weight	Overweight	Obesity	Total
	n (%)	n (%)	n (%)	n (%)	n	n (%)	n (%)	n (%)	n (%)	n
**Children <5 years**	14 (2.3)	2 (0.3)	55 (9.0)^a^	31(5.1)^a^	612	16 (2.6)	4 (0,^7^)	39 (6.3)^a^	29 (4.7)^a^	615
**Children ≥5 years**	6 (1.4)	2 (0.5)	67 (16.1)^a^	42 (10.1)^a^	416	5 (1,3)	5 (1,3)	70 (17.9)^a^	43 (11.0)^a^	390
Girls	9 (1.7)	2 (0.4)	61(11.7)	32 (6.2)	520	15 (2.9)	3 (0.6)	43 (8.3)^b^	36 (6.9)	519
Boys	11 (2.2)	2 (0.4)	61 (12.0)	41 (8.1)	508	6 (1.2)	6 (1.2)	66 (13.6)^b^	36 (7.4)	486
Total	20 (1.9)	4 (0.4)	122 (11.9)	73 (7.1)	1028	21 (2.1)	9 (0.9)	109 (10.8)	72 (7.2)	1005

^a^The frequency differs significantly according to age group within the same period; ^b^The frequency differs significantly according to sex within the same period.

**Figure 1:**
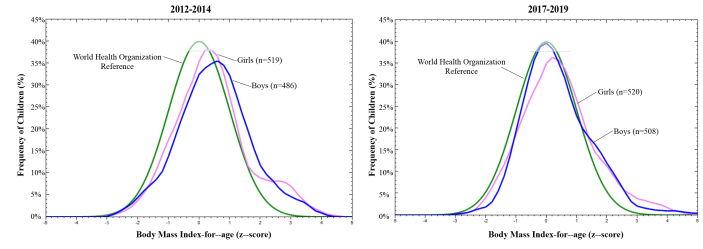
Distribution of body mass index-for-age, according to sex of preschool children in public schools. Macaé, 2012-2014 (n=1,028) and 2017-2019 (n=1,005)

The prevalence of body mass index-for-age deficit was less than 1.0% in both periods. The frequency of overweight reached 11.9% at moment 1 and 10.8% at moment 2. The frequency of obesity reached 7.1% at moment 1 and 7.2% at moment 2, with no statistical difference between the time points (Table 1). The frequencies of overweight and obesity were higher among preschoolers aged 5 years or older (p-value<0.010). At moment 2, boys had a significantly higher prevalence of overweight (13.6%) than girls (8.3%) (Table 1). The distribution of body mass index-for-age showed a shift to the right in relation to the reference curve, in both periods (Figure 1).

## Discussion

The frequencies of overweight and obesity were high in this study, while the prevalence of nutritional deficits was low at both surveys. Overweight and obesity were more common among children over 5 years old and among boys, which resulted in a profile like that observed in Southeast Brazil (5,6). 

The prevalence of height deficit was lower than national estimates. Data from 2019 on children under 5 years old across Brazil estimated a 5.2% rate for height deficit (5), that is, more than double the frequency observed among preschool children in Macaé. Among the measures to address the height deficit, the following stand out: increasing the purchasing power of the poorest classes, increasing maternal education and access to health and sanitation (22). 

Compared to other Brazilian municipalities, health, and sanitation coverage in Macaé was relatively better (10.11), which is consistent with a small height deficit. Although the sample evaluated is not large enough to accurately estimate the low point prevalence of this deviation, if a substantial increase had occurred, it would have been possible to detect it at moment 2.

Among children under 5 years of age, the pooled prevalence of overweight plus obesity was greater than 10.0% at both surveys, i.e., approximately twice the value expected in the reference population (4). A similar prevalence (10.5%) was observed in Brazilian children of the same age in the Southeast in 2019 (5). Among older preschoolers, the pooled prevalence of overweight and obesity was approximately 10.0% higher in Macaé than that predicted in the reference population (21). National data from 2008-2009 for children aged 5 to 9 years indicated a higher frequency (33.5%) than those in this study (6). 

The most notable result of this study was the stability in the frequency of nutritional deviations after the five-year frame. This finding did not follow the national trend of the nutritional transition process, which is to reduce deficits and increase nutritional excess over time (5,6,22).

This divergence was even more unexpected given the adverse economic context. The panel interval was marked by the global economic crisis in the oil sector in 2014 (10), and by national economic austerity measures and undermining of public policies in 2016 (23), which impacted Macaé’s economy. As there was a worsening of health indicators in the municipality during this period (11), it was expected that anthropometric indicators would also deteriorated. It should be said that the supply of public-school meals in Macaé did not undergo any changes during the panel period and, therefore, did not constitute an interfering factor in the indicators. There was a broad change in terms of educational actions, as the period coincided with the publication of the Dietary Guidelines (8) and its use in municipal schools, which may have contributed to mitigating the negative impacts of the crisis, by promoting healthy eating and the adequacy of children’s nutritional status. 

The expansion of public universities to the smaller municipalities induces social transformations (24). Originally a fishing town, Macaé underwent an abrupt and disorderly growth due to the oil exploration in the 1970s, becoming known as the “national oil capital” (10). Around 2010, the city began to encompass higher education institutions that have gradually increased Macaé’s potential beyond the oil industry, which also encourages the title of “city of knowledge”. This is an intricated sociopolitical and health framework, which has strengthened partnerships between different social actors and the production of knowledge. These partnerships can contribute to effective actions and allocation of public resources (20,25).

University extension helps building these partnerships, as it provides for interprofessional and dialogic interaction between academia, local services, and society (26). Through research-action (27), extension in Macaé may be contributing to intersectoral policies, such as the PNAE, by developing educational actions supported by the Dietary Guidelines (8).

Educational activities, such as those implemented in Macaé, promote participants’ awareness and support healthier food choices (13,14). Besides, these activities encourage family members and educators to use the Dietary Guidelines and promote healthy eating at school. Educators have strong potential to drive social change, since they spread knowledge to many. When families get involved, school-based actions can also influence eating habits at home, reinforcing what children learn at school (19).

As of 2018, food and nutrition education became a required topic of Brazilian basic education (28).Complying with this legislation has been a challenge for teaching teams (29). The Dietary Guidelines can encourage these teams to implement healthy eating in schools. The joint actions developed by government programs and municipality may favor the adequate nutritional status of preschoolers or, at least, prevent the worsening of nutritional deviations.

One of the strengths of this study is the nutritional assessment carried out on panels based on primary data, with a large sample, having as a time frame the implementation of educational actions based on the Dietary Guidelines. Besides, the panel interval encompass a scenario of economic vulnerability, different from the Brazilian capitals, where most of the research on nutrition in the country is concentrated. Notably this study is the result of the articulation between academia and the municipal public sector, using data from the education service, which is rarely publicized.

Given its link to the service, this work included only a few variables and a non-probabilistic sample, which limits the analysis of determinants of nutritional status and the generalization of the findings. The inclusion of schools from different socioeconomic regions helped to mitigate external validity limitations. Given the sample size and estimated prevalence, the study had high precision in detecting overweight and obesity, which ensured the internal validity of comparisons between periods. On the other hand, it is not possible to ensure that nutritional deficits were estimated with adequate precision, because the observed prevalences were lower than those estimated for the Southeast and Brazil (5). A significantly larger sample size would be needed to detect such a rare event.

This study sheds light on the limits and potential of integrating university and services. Although the academy supports the municipality and promotes learning for all involved, it is not up to it and it would be unable to replace the service in implementing the PNAE. Through university extension, this study highlighted the relevance of the School Nutrition Coordination and the PNAE. This study also points to the need for municipal managers to reconsider the allocation of resources to strengthen the service. 

This work confirmed the importance of monitoring anthropometric indicators, as these vary depending on the local situation. The data from moment 2 were collected before the health crisis caused by the COVID-19 pandemic, which aggravated the economic recession, health disparities and food insecurity. On-site classes and schools meals were suspended, as well as university extension activities (9,30). These changes combined with the interruption of actions, may lead to a higher frequency of nutritional deviations. 

The direction of these deviations may be either deficit or excess, as these may be correlated (2). Therefore, intersectoral policies and actions, involving different social actors and their management levels are important to maintain food and nutritional surveillance and promote adequate nutritional status (15,20). This study can contribute to ensuring that such policies and actions are assertive and contextualized with the municipal reality. These reflections from Macaé may inspire other locations to monitor their child nutritional indicators, incorporate the Dietary Guidelines into educational activities and recognize the potential of these activities in protecting children’s health. 

## Data Availability

The database of the first survey is not publicly available, as it was used under license from the Municipal Department of Education of Macaé for this study. The second database and analysis codes used in this study are available at https://www.nesane.net/.
