# Supplementary material for: Nutritional status of preschoolers in the public school system of Macaé: a panel study conducted between 2012-2014 and 2017-2019 in the context of the oil crisis and the implementation of educational actions based on the Dietary Guidelines for the Brazilian Population
Source: Epidemiol Serv Saude. 2025 Aug 4;34:e20240386. doi: 10.1590/S2237-96222025v34e20240386.en (PMC12338992; doi:10.1590/S2237-96222025v34e20240386.en)
Supplement: Translation

# Translation: Estado nutricional de pré-escolares da rede pública de Macaé: estudo de painéis realizado entre 2012-2014 e 2017-2019 no contexto de crise petrolífera e de implementação de ações educativas pautadas no Guia Alimentar para a População Brasileira

**DOI:** 10.1590/S2237-96222025v34e20240386.pt

**Authors:** Ana Eliza Port Lourenço, Thácia de Araújo Amado Coutinho, Luana Silva Monteiro, Laís Vargas Botelho, Priscila Vieira Pontes, Naiara Sperandio

**Affiliations:** 1 Universidade Federal do Rio de Janeiro, Instituto de Alimentação e Nutrição, Macaé, RJ, Brasil

**Keywords:** Antropometria, Pré-Escolar, Inquéritos Epidemiológicos, Guias Alimentares, Serviços de Saúde Escolar

## Abstract

**Objetivo:**

Avaliar o estado nutricional de pré-escolares da rede pública de Macaé, Rio de Janeiro, em dois momentos, separados por intervalo de cinco anos.

**Métodos:**

Trata-se de estudo de painéis realizados entre 2012-2014 e 2017-2019, incluindo cinco escolas, localizadas em regiões com diferentes condições socioeconômicas. As amostras abrangeram 15,0% do total de alunos da rede pública de educação infantil de Macaé. Avaliaram-se os indicadores estatura-para-idade e índice de massa corporal-para-idade. Foram calculadas medidas resumo de variáveis antropométricas, com estratificação por sexo e faixa etária. Para comparações entre grupos e inquéritos, foram utilizados os testes t de Student ou Mann-Whitney e qui-quadrado.

**Resultados:**

Em cada período, foram avaliados 1.028 e 1.005 pré-escolares. A média de idade foi 55,3 e 54,7 meses, sendo 50,6% e 51,6% meninas. Identificou-se déficit de estatura em 1,9% e 2,1% das crianças, sem diferença significativa por sexo, faixa etária ou entre inquéritos. A prevalência de obesidade foi 5,1% e 4,7% entre os menores de 5 anos, e 10,1% e 11,0% entre os mais velhos, também sem diferença entre os inquéritos.

**Conclusão:**

Nos dois momentos do painel, a prevalência de déficit nutricional foi baixa, e de obesidade, relativamente alta. Apesar do contexto de crise na economia local, a frequência dos indicadores antropométricos não deteriorou entre os momentos. A implementação de ações educativas nas escolas, orientadas pelo *Guia Alimentar para a População Brasileira*, pode ter contribuído na proteção da saúde das crianças.

**Aspectos éticos**

A presente pesquisa respeitou os princípios éticos, obtendo os seguintes dados de aprovação:

Comitê de ética em pesquisa: Universidade Federal do Rio de Janeiro

Número do parecer: 1.319.805

Data de aprovação: 17/11/2015

Certificado de apresentação de apreciação ética: 49258313.1.0000.5257

Registro do consentimento livre e esclarecido: Obtido de todos os participantes antes da coleta do inquérito ocorrido de 2016 a 2019.

Observações: Os dados do inquérito ocorrido de 2012 a 2014 tinham curadoria da Secretaria Municipal de Educação de Macaé e foram cedidos à professora Ana Eliza Port Lourenço para estudo.

## Introdução

O estado nutricional pode ser entendido como o resumo da relação entre indivíduos, alimentos e ambiente. Quando há desequilíbrio nessa relação, ocorrem desvios nutricionais que trazem riscos à saúde (1). Tal desequilíbrio pode acontecer devido ao mosaico de determinantes, tanto proximais quanto mais distais aos indivíduos, que interferem no processo de transição nutricional nas sociedades (2). Monitorar o estado nutricional de coletividades é fundamental, especialmente na infância, pois permite avaliar o crescimento, o ganho de peso e o desenvolvimento das crianças, além de auxiliar no direcionamento de políticas, programas e ações voltadas à saúde (3).

Os indicadores antropométricos são úteis, visto que identificam os desvios nutricionais e refletem as condições gerais de saúde das sociedades (4). A vigilância alimentar e nutricional na Atenção Primária à Saúde e as pesquisas para acompanhamento da transição nutricional no Brasil exemplificam a potencialidade de aplicação desses indicadores (5-7). Também é pertinente utilizá-los em estudos, de larga ou menor escala, que visem avaliar tendências a partir de adventos político-sociais, como o surgimento da classificação de alimentos conforme a natureza, a extensão e o propósito do processamento, expressa no *Guia Alimentar para a População Brasileira* (8); ou de circunstâncias inesperadas, como a pandemia de covid-19 (9) e outras situações de crise.

Pesquisas de pequena escala são relevantes, pois acontecimentos em nível global ou nacional podem repercutir de maneira distinta nas diferentes conjunturas locais. Em menor escala, é possível analisar detalhes do contexto nutricional que poderiam não ser perceptíveis por um olhar macro. A crise mundial no ramo petrolífero, ocorrida em 2014, impactou, de maneira drástica, os municípios brasileiros dessa base econômica, como Macaé, situado no norte do estado do Rio de Janeiro. Macaé apresenta um dos maiores valores de produto interno bruto per capita do país, porém, em decorrência da crise, houve desinvestimento no município, com prejuízos para a saúde e qualidade de vida (10,11).

Outra particularidade de Macaé é que, mesmo sendo um município interiorano de porte médio, abarca quatro universidades públicas. A vivacidade dos projetos de pesquisa e extensão universitária favorece a construção de vínculos com os serviços locais e a sociedade. Ressaltam-se especialmente projetos na temática da promoção da saúde e da alimentação saudável (12-14).

A partir da publicação, em 2014, o *Guia Alimentar para a População Brasileira* passou a ser o material de referência desses projetos, que atuavam em diferentes espaços, sobretudo nas escolas, por serem locais favoráveis para a materialização de ações intersetoriais, aproximando saúde e educação (15). As diretrizes do Guia passaram a ser aplicadas em consonância com o Programa Nacional de Alimentação Escolar (PNAE) e o Programa Saúde na Escola, o que favoreceu hábitos saudáveis de crianças e educadores (16). 

O objetivo deste estudo foi avaliar o estado nutricional de pré-escolares da rede pública de Macaé, Rio de Janeiro, em dois momentos, separados por um intervalo de cinco anos. Teve-se por hipótese que, em razão da crise petrolífera, seria observada piora na frequência das diferentes formas de má nutrição infantil.

## Métodos

### Delineamento

Trata-se de estudo observacional, de painéis, ocorrido em dois momentos: de maio de 2012 a julho de 2014 (momento 1) e de maio de 2017 a dezembro de 2019 (momento 2). O estudo analisou dados primários, coletados em escolas públicas de educação infantil, via uma parceria entre a Universidade Federal do Rio de Janeiro e o serviço municipal de educação de Macaé. O intervalo de avaliação antropométrica dos momentos 1 e 2 foi de aproximadamente cinco anos em cada escola.

### Contexto: articulação universidade-serviço na operacionalização do PNAE

A rede pública municipal de educação de Macaé contava com 107 escolas, que atendiam 40 mil estudantes (17). A Coordenadoria de Nutrição Escolar da Secretaria Municipal de Educação de Macaé era responsável pela execução do PNAE na esfera municipal. Além de ofertar alimentação aos estudantes, o PNAE prevê a avaliação antropométrica e a realização de atividades de educação alimentar e nutricional, embasadas no diagnóstico nutricional (18). 

No período deste estudo, a equipe da Coordenadoria de Nutrição Escolar era composta por quatro nutricionistas, número insuficiente para atender às demandas da rede e operacionalizar plenamente as diretrizes do PNAE. A gestão da alimentação escolar e o planejamento de cardápios eram feitos pela coordenadoria, em conformidade com a legislação vigente, enquanto a execução dos cardápios cabia a uma empresa terceirizada. Atividades antropométricas e educativas eram escassas. Para enfrentar essa limitação e potencializar a cobertura das ações, foi estabelecida a parceria entre a coordenadoria e um núcleo de estudos da referida universidade (14), que atuou de forma integrada ao serviço, desde 2011.

Essa articulação ocorreu por meio de projetos de extensão desse núcleo, que colaboravam diretamente na rotina do serviço, visando alcançar as metas previstas pelo PNAE. Este estudo é fruto de tal aproximação universidade-serviço, em que docentes e graduandos integram-se a nutricionistas do município, formando uma equipe única. Embora seja estudo primário, a coleta de dados seguiu o modus operandi habitual da Coordenadoria de Nutrição Escolar. A logística de campo e as variáveis coletadas não foram definidas para atender especificamente aos objetivos de um estudo. Pelo contrário, a realização deste estudo representou a concretização dos objetivos da coordenadoria e, em última instância, do PNAE. 

Em cada escola, eram feitas: uma visita de planejamento; duas a três visitas para medições antropométricas; uma visita para compartilhar resultados e planejar atividades educativas; e visitas para realização das atividades, sendo de duas a três com as crianças e uma a duas com educadores e famílias, dependendo do tamanho da escola. Além da equipe da coordenadoria e da universidade, profissionais de saúde do território adscrito colaboravam nas ações educativas, integrando com o Programa Saúde na Escola. 

As ações eram guiadas pelo Marco de Referência de Educação Alimentar e Nutricional para Políticas Públicas (19) e pelo Guia (8). A metodologia educativa utilizada encontra-se detalhada em publicações anteriores (13,14,20). Em linhas gerais, as atividades com crianças trabalhavam de maneira lúdica as orientações do Guia sobre: evitar ultraprocessados; basear a dieta em alimentos in natura ou minimamente processados; e desenvolver habilidades culinárias. Com os educadores e as famílias, além de orientações gerais do guia alimentar, as ações visavam promover reflexão sobre o princípio de autonomia nas escolhas alimentares.

### Fontes de dados e mensuração

Os dados dos momentos 1 e 2 foram coletados no âmbito do serviço da Coordenadoria de Nutrição Escolar, em parceria com o núcleo de estudos da universidade. O núcleo proporcionou os equipamentos de coleta e o treinamento prévio da equipe, que contou com nutricionistas da coordenadoria e estudantes universitários. 

Em ambos os momentos, a avaliação antropométrica ocorreu conforme previsto na Atenção Primária em Saúde (7), no horário escolar, com as crianças vestidas com o uniforme da escola. O peso foi aferido em quilogramas, utilizando balança eletrônica, da marca Tanita, com precisão de 100 g. A estatura foi aferida em centímetros, duas vezes consecutivas para cada criança, utilizando estadiômetro de madeira, marca Alturaexata, com precisão de 1 mm. Para fins de análise, utilizou-se a média das duas medidas. Informações sobre sexo e data de nascimento foram obtidas em documentos da escola. A idade foi calculada pela diferença entre as datas da aferição e de nascimento.

### Participantes

Em ambos os momentos, foi avaliada uma amostra não probabilística, composta por pré-escolares de 2 a 7 anos incompletos, matriculados em cinco escolas de área urbana. Estas foram selecionadas por conveniência pela Coordenadoria de Nutrição Escolar, que, por dificuldades logísticas, priorizava unidades com maior quantitativo de alunos e fácil acesso por transporte público. 

Na área urbana de Macaé, havia 45 escolas públicas de educação infantil, totalizando 8 mil pré-escolares. A cidade é subdividida em setores administrativos, que podem ser agrupados em três regiões socioeconômicas. Na região mais pobre, onde há crescimento urbano desordenado e saneamento precário, situavam-se duas das escolas avaliadas. Outra escola estava na região socioeconômica intermediária, característica do centro de Macaé. As outras duas estavam na parte litorânea, caracterizada por estabelecimentos comerciais variados, turismo e residências de alto padrão.

Como critérios de inclusão, foram considerados todos os pré-escolares regularmente matriculados nas escolas selecionadas. Foram excluídos estudantes com condições de saúde mental ou física que interferissem na avaliação antropométrica. 

No momento 1, dos 1.209 pré-escolares matriculados, 24 foram excluídos e 157 foram perdidos, sendo analisados 1.028 pré-escolares (85,0% dos elegíveis). No momento 2, das 1.116 crianças matriculadas nas mesmas escolas, 8 foram excluídas, 103 perdidas e 1.005 analisadas (90,0% das elegíveis).

### Variáveis 

As variáveis numéricas analisadas foram aquelas previstas no PNAE (peso, estatura e idade), bem como os índices antropométricos estatura-para-idade e índice de massa corporal-para-idade. Esses índices foram expressos em escores-z, calculados conforme as curvas de referência da Organização Mundial da Saúde (OMS) (4,21), utilizando o programa WHO Anthro Plus (v1.0.4).

Tais índices também foram categorizados. Crianças com escore-z <-2 desvios-padrão para estatura-para-idade ou para índice de massa corporal-para-idade foram consideradas com déficit nesses indicadores. Crianças menores de 5 anos foram classificadas com sobrepeso, quando o índice de massa corporal-para-idade era ≥2 desvios-padrão e <3 desvios-padrão, e com obesidade, quando era ≥3 desvios-padrão. Os pré-escolares mais velhos foram considerados com sobrepeso quando o índice de massa corporal-para-idade era ≥1 desvio-padrão e <2 desvios-padrão, e com obesidade, quando era ≥2 desvios-padrão (7). 

### Controle de viés

Os procedimentos de coleta de dados foram os mesmos em ambos os momentos, bem como os indicadores analisados, o que favoreceu a comparação. Para evitar perda de participantes, foram realizadas mais de uma visita em cada escola, em dias da semana diferentes e não consecutivos.

Para minimizar erros, utilizaram-se as informações de sexo e data de nascimento registradas em documento da escola. A medição de estatura ocorreu em duplicata. 

Por não ser probabilística, a amostra pôde sofrer viés de seleção. Ao incluir escolas de diferentes regiões socioeconômicas, buscou-se mitigar esse viés e melhorar a validade externa.

### Tamanho do estudo 

Em ambos os momentos, o quantitativo de crianças da amostra abrangeu cerca de 15,0% do total de pré-escolares da área urbana e dos que estudavam em cada uma das três regiões socioeconômicas de Macaé.

### Métodos estatísticos

Realizou-se análise descritiva das variáveis antropométricas e dos desvios nutricionais segundo período (momento 1, momento 2), sexo (masculino, feminino) e faixa etária, considerando pré-escolares com idade inferior a 5 anos e aqueles com 5 anos ou mais. As variáveis categóricas foram sumarizadas em frequências absolutas e relativas, e as contínuas, em médias e desvios-padrão ou medianas e intervalo interquartílico, dependendo da distribuição dos dados. As comparações entre grupos para variáveis contínuas foram realizadas usando o teste t de Student ou de Mann-Whitney. Para as variáveis categóricas, utilizou-se o teste qui-quadrado. 

Análises de poder foram conduzidas para avaliar a capacidade do estudo de identificar diferenças reais nas prevalências dos desvios nutricionais entre os dois momentos do painel, em cada faixa etária, considerando poder estatístico mínimo de 80,0%. Observou-se que a amostra obtida em ambos os momentos possuía poder estatístico de 100,0% para detectar a prevalência de sobrepeso e obesidade entre menores de 5 anos e entre as crianças mais velhas. Porém, o poder amostral não era capaz de detectar diferenças nas frequências de déficits de estatura-para-idade e de índice de massa corporal-para-idade.

Adotou-se nível de significância de 5,0% em todas as análises. O Statistical Package for the Social Sciences, versão 19, foi o programa estatístico utilizado.

## Resultados

Entre os 1.028 avaliados no momento 1, 50,6% eram do sexo feminino, 59,5% eram menores de 5 anos, e a média geral de idade foi 55,3 meses. Entre os 1.005 participantes do momento 2, 51,6% eram meninas, 61,2% eram menores de 5 anos, e a média geral de idade foi 54,7 meses (Tabelas 1 e 2).

As médias de estatura-para-idade foram próximas a 0 (Tabela 2). A curva de distribuição se assemelhou ao padrão da OMS nos momento 1 e 2. Entre os meninos, a distribuição de estatura-para-idade apresentou valores menores no momento 2 (p-valor 0,020). A prevalência de déficit de estatura-para-idade foi 1,9% no momento 1 e 2,1% no momento 2, sem diferença significativa segundo sexo, faixa etária ou entre momentos (Tabela 1). 

**Tabela 2:** Estatística descritiva das variáveis antropométricas e idade, segundo faixa etária e sexo de pré-escolares da rede pública. Macaé, 2012-2014 (n=1.028) e 2017-2019 (n=1.005)

	2012-2014					2017-2019				
	Idade (meses)	Estatura (cm)	Peso (kg)	Estatura-para-idade (escore-z)	Índice de massa corporal-para-idade (escore-z)	Idade (meses)	Estatura (cm)	Peso (kg)	Estatura-para-idade (escore-z)	Índice de massa corporal-para-idade (escore-z)
	Média (desvio-padrão)	Mediana (intervalo interquartílico)				Média (desvio-padrão)	Mediana (intervalo interquartílico)			
**Crianças <5 anos**	46,5 (8,8)	102,5 (97,5; 106,8)	16,7 (14,9; 19,0)	-0,02 (-0,71; 0,77)	0,61 (-0,15; 1,39)	46,0 (8,9)	101,1 (95,8; 106,8)	16,4 (14,6; 18,6)	-0,12 (-0,86; 0,63)	0,56 (-0,04; 1,26)
**Crianças** ≥**5 anos**	68,2 (4,9)	114,1 (110,3; 118,0)	20,4 (18,7; 23,0)	0,13 (-0,54; 0,78)	0,28 (-0,39; 1,06)	68,4 (5,0)	113,8 (110,3; 117,5)	20,5 (18,6; 22,9)	-0,01 (-0,61; 0,71)	0,44 (-0,31; 1,11)
Meninas	55,3 (12,8)	106,8 (100,4; 113,4)	18,2 (15,6; 20,9)	-0,02 (-0,60; 0,71)	0,47 (-0,31; 1,21)	54,4 (13,3)	106,9 (98,9; 112,0)	18,0 (15,4; 20,3)	-0,10 (-0,74; 0,64)	0,45 (-0,21; 1,14)
Meninos	55,3 (13,2)	107,9 (101,5; 113,9)	18,6 (16,4; 21,1)	0,12^a^ (-0,64; 0,83)	0,52 (-0,27; 1,34)	55,0 (13,3)	107,3 (100,0; 114,0)	18,3 (16,0; 21,2)	-0,04^a^ (-0,82; 0,68)	0,57 (-0,03; 1,24)
Total	55,3 (13,0)	107,4^a^ (100,9; 113,6)	18,3 (16,0; 21,0)	0,03^a^ (-0,62; 0,77)	0,48 (-0,28; 1,29)	54,7 (13,3)	107,1^a^ (99,4; 112,9)	18,1 (15,7; 20,8)	-0,08^a^ (-0,78; 0,66)	0,52 (-0,14; 1,20)

^a^A distribuição da variável difere significativamente entre os períodos.

**Tabela 1:** Prevalência de desvios nutricionais, segundo faixa etária e sexo de pré-escolares da rede pública. Macaé, 2012-2014 (n=1.028) e 2017-2019 (n=1.005)

	2012-2014					2017-2019				
	Baixa estatura	Magreza	Sobrepeso	Obesidade	Total	Baixa estatura	Magreza	Sobrepeso	Obesidade	Total
	n (%)	n (%)	n (%)	n (%)	n	n (%)	n (%)	n (%)	n (%)	n
**Crianças <5 anos**	14 (2,3)	2 (0,3)	55 (9,0)^a^	31(5,1)^a^	612	16 (2,6)	4 (0,7)	39 (6,3)^a^	29 (4,7)^a^	615
**Crianças** ≥**5 anos**	6 (1,4)	2 (0,5)	67 (16,1)^a^	42 (10,1)^a^	416	5 (1,3)	5 (1,3)	70 (17,9)^a^	43 (11,0)^a^	390
Meninas	9 (1,7)	2 (0,4)	61(11,7)	32 (6,2)	520	15 (2,9)	3 (0,6)	43 (8,3)^b^	36 (6,9)	519
Meninos	11 (2,2)	2 (0,4)	61(12,0)	41(8,1)	508	6 (1,2)	6 (1,2)	66 (13,6)^b^	36 (7,4)	486
Total	20 (1,9)	4 (0,4)	122 (11,9)	73 (7,1)	1028	21 (2,1)	9 (0,9)	109 (10,8)	72 (7,2)	1005

^a^A frequência difere significativamente segundo faixa etária dentro do mesmo período; ^b^A frequência difere significativamente segundo sexo dentro do mesmo período.

A prevalência de déficit de índice de massa corporal-para-idade foi inferior a 1,0% nos dois períodos. A frequência de sobrepeso alcançou 11,9% no momento 1 e 10,8% no momento 2. A frequência de obesidade alcançou 7,1% no momento 1 e 7,2% no momento 2, sem diferença estatística entre os momentos (Tabela 1). As frequências de sobrepeso e de obesidade foram maiores entre pré-escolares com 5 anos ou mais (p-valor<0,010). No momento 2, os meninos apresentaram prevalência de sobrepeso (13,6%) significativamente maior do que as meninas (8,3%) (Tabela 1). A distribuição de índice de massa corporal-para-idade apresentou desvio para a direita em relação à curva de referência, em ambos os períodos (Figura 1).

**Figura 1:** Distribuição do índice de massa corporal-para-idade, segundo sexo de pré-escolares da rede pública. Macaé, 2012-2014 (n=1.028) e 2017-2019 (n=1.005)

## Discussão

As frequências de sobrepeso e de obesidade foram elevadas neste estudo, enquanto a prevalência de déficits nutricionais foi baixa em ambos os momentos avaliados. Sobrepeso e obesidade estavam mais presentes entre crianças maiores de 5 anos e entre meninos, o que configurou perfil semelhante ao observado no Sudeste brasileiro (5,6). 

A prevalência de déficit de estatura foi inferior às estimativas nacionais. Dados de 2019 sobre menores de 5 anos de todo o Brasil estimavam 5,2% de déficit de estatura (5), ou seja, mais que o dobro da frequência observada nos pré-escolares de Macaé. Entre as medidas para enfrentar o déficit estatural, destacam-se a ampliação do poder aquisitivo das camadas mais pauperizadas, a elevação da escolaridade materna e a universalização do acesso à saúde e ao saneamento (22). 

Em comparação a outros municípios brasileiros, a cobertura de saúde e saneamento em Macaé era relativamente melhor (10,11), o que condiz com déficit estatural diminuto. Embora a amostra avaliada não seja grande o suficiente para estimar com precisão a baixa prevalência pontual desse desvio, caso um aumento substancial tivesse ocorrido, seria possível detectá-lo no momento 2.

Entre os menores de 5 anos, a prevalência agrupada de sobrepeso mais obesidade foi superior a 10,0% em ambos os momentos, ou seja, cerca do dobro do valor esperado na população de referência (4). Prevalência semelhante (10,5%) foi observada nas crianças brasileiras da mesma idade no Sudeste em 2019 (5). Entre os pré-escolares mais velhos, a prevalência agrupada de sobrepeso e obesidade foi aproximadamente 10,0% mais elevada em Macaé do que a prevista na população de referência (21). Dados nacionais de 2008-2009 para crianças de 5 a 9 anos indicaram frequência mais elevada (33,5%) do que as deste estudo (6). 

O resultado de maior destaque deste estudo foi a estabilidade na frequência de desvios nutricionais após o período de cinco anos. Tal achado não seguiu a tendência nacional do processo de transição nutricional, que é de redução de déficit e aumento do excesso nutricional ao longo do tempo (5,6,22).

Essa divergência era ainda mais inesperada diante do contexto econômico adverso. O intervalo do painel foi marcado pela crise econômica global no setor petrolífero em 2014 (10), agravada por medidas nacionais de austeridade econômica e desmonte de políticas públicas em 2016 (23), que impactou a economia de Macaé. Como houve recrudescimento de indicadores de saúde no município nesse período (11), era esperado que os indicadores antropométricos também se agravassem. Cabe dizer que a oferta da alimentação escolar pública em Macaé não sofreu modificações durante o período do painel e, portanto, não se configurou como fator de interferência nos indicadores. Houve ampla mudança no que se refere às ações educativas, pois o período coincidiu com a publicação do Guia (8) e sua utilização nas escolas municipais, o que pode ter contribuído para mitigar os impactos negativos da crise, ao favorecer a alimentação saudável e a adequação do estado nutricional infantil. 

Destaca-se o potencial da interiorização das universidades públicas (24) como indutora de transformações. De origem pesqueira, Macaé passou por expansão abrupta e desordenada a partir da exploração petrolífera iniciada na década de 1970, tornando-se conhecida como a “capital nacional do petróleo” (10). Por volta de 2010, o município passou a abrigar instituições de ensino superior que, paulatinamente, vêm ampliando o potencial de Macaé para além da indústria petrolífera, o que também motiva o título de “cidade do conhecimento”. Trata-se de intrincado quadro sociopolítico e de saúde, que tem estreitado parcerias entre atores sociais diversos e fortalecido a produção de conhecimento. Essas parcerias podem contribuir para ações eficazes e adequada alocação de recursos públicos (20,25).

A extensão universitária é uma via privilegiada para a construção dessas parcerias, visto que prevê a interprofissionalidade e a interação dialógica entre academia, serviços locais e sociedade (26). Por meio da pesquisa-ação (27), a extensão em Macaé pode estar contribuindo para materializar políticas intersetoriais, como o PNAE, ao realizar ações educativas respaldadas no *Guia Alimentar para a População Brasileira* (8).

Mais que sensibilizar os participantes e dar suporte às escolhas alimentares, atividades educativas, como as implementadas em Macaé (13,14), incentivam familiares e educadores a utilizar o guia alimentar e a atuar na promoção da alimentação saudável na escola. O potencial de transformação social é vasto, considerando que os educadores são multiplicadores de conhecimento e que ações com as famílias podem reverberar nas práticas alimentares domésticas, o que reforça o processo educativo iniciado no espaço escolar (19).

A partir de 2018, a educação alimentar e nutricional tornou-se parte integrante do currículo da educação básica no Brasil (28), e atender a essa legislação tem se mostrado um desafio para as equipes pedagógicas (29). Conhecer o Guia como referência pode estimular o protagonismo dessas equipes na efetivação da alimentação saudável nas escolas. É possível que as ações conjuntas desenvolvidas pelos programas governamentais e pelo município estejam favorecendo o adequado estado nutricional dos pré-escolares ou, ao menos, evitando o agravamento dos desvios nutricionais. 

Entre os pontos fortes deste estudo, está a avaliação nutricional realizada em painéis baseados em dados primários, com ampla amostra, tendo como marco temporal a implementação de ações educativas baseadas no Guia. Acrescenta-se o cenário peculiar de vulnerabilidade econômica, diferente do contexto de capitais brasileiras, onde se concentra grande parte das pesquisas sobre nutrição no país. É marcante também que este estudo resulta da articulação entre academia e setor público municipal, utilizando dados do serviço de educação, que raramente são publicizados.

Justamente pela indissociabilidade com o serviço, este trabalho incluiu número reduzido de variáveis e baseou-se em amostra não probabilística, o que limita a análise de determinantes do estado nutricional e a generalização dos achados. A inclusão de escolas de diferentes regiões socioeconômicas ajudou a mitigar limitações de validade externa. Dado o tamanho das amostras e as prevalências estimadas, o estudo possuiu alta precisão para detectar sobrepeso e obesidade, o que assegurou a validade interna das comparações entre os períodos. Por outro lado, não é possível assegurar que os déficits nutricionais tenham sido estimados com precisão adequada, porque as prevalências observadas foram inferiores às estimadas para o Sudeste e o Brasil (5). Seria necessário um tamanho amostral significativamente maior para detectar evento tão raro.

Da experiência deste estudo emergem reflexões sobre limites e potencialidades da integração universidade-serviço. Embora a academia siga apoiando o município e favorecendo o aprendizado de todos os envolvidos, não cabe a ela (nem seria capaz) substituir o serviço na concretização do PNAE. Por meio da extensão universitária, este estudo explicitou o potencial e a relevância das ações da Coordenadoria de Nutrição Escolar e do PNAE. Apontou-se também a necessidade de os gestores municipais reconsiderarem a alocação de recursos para fortalecer o serviço. 

Este trabalho ratificou a importância do monitoramento dos indicadores antropométricos, já que esses podem oscilar a depender da situação local. Vale enfatizar que os dados do momento 2 foram coletados antes da crise sanitária provocada pela pandemia de covid-19, que acirrou a recessão econômica, as iniquidades em saúde e a insegurança alimentar no país. Houve suspensão das aulas presenciais e da oferta da alimentação escolar, bem como das atividades de extensão universitária (9,30). Tal inesperada transformação nas condições de vida, somada à descontinuação de ações, pode provocar aumento da frequência de desvios nutricionais. 

A direção desses desvios pode ser tanto de déficit quanto de excesso, já que esses podem estar correlacionados (2). Essa complexidade reforça a relevância de políticas e ações intersetoriais, envolvendo diferentes atores sociais e seus níveis de gerência, para manter a vigilância alimentar e nutricional e promover a adequação do estado nutricional (15,20). Este estudo pode contribuir para que tais políticas e ações sejam desenvolvidas de maneira assertiva e contextualizada com a realidade municipal. Essas reflexões sobre Macaé podem motivar outras localidades a acompanharem seus indicadores nutricionais infantis, incorporarem o Guia nas atividades educativas nas escolas e reconhecerem o potencial dessas atividades na proteção da saúde das crianças. 

## Data Availability

O banco de dados do primeiro inquérito não está disponível publicamente, pois foram utilizados sob licença da Secretaria Municipal de Educação de Macaé para este estudo. O segundo banco de dados e códigos de análise utilizados na pesquisa estão disponíveis em: https://www.nesane.net/.
